# *SMG1* heterozygosity exacerbates haematopoietic cancer development in *Atm* null mice by increasing persistent DNA damage and oxidative stress

**DOI:** 10.1111/jcmm.14685

**Published:** 2019-09-29

**Authors:** Uda Ho, John Luff, Alexander James, Cheok Soon Lee, Hazel Quek, Hui‐Chi Lai, Simon Apte, Yi Chieh Lim, Martin F. Lavin, Tara L. Roberts

**Affiliations:** ^1^ School of Biomedical Sciences Faculty of Medicine University of Queensland St Lucia Qld Australia; ^2^ UQCCR University of Queensland Brisbane Qld Australia; ^3^ The Ingham Institute for Applied Medical Research and School of Medicine Western Sydney University Liverpool NSW Australia; ^4^ South West Sydney Clinical School UNSW Sydney Liverpool NSW Australia; ^5^ Department of Anatomical Pathology Molecular Pathology Laboratory Liverpool Hospital Liverpool NSW Australia; ^6^ QIMR Berghofer Medical Research Institute Herston Qld Australia; ^7^ Danish Cancer Society Research Centre Copenhagen Denmark

**Keywords:** cancer, DNA damage, inflammation, lymphoma, oxidative stress

## Abstract

Suppressor of morphogenesis in genitalia 1 (SMG1) and ataxia telangiectasia mutated (ATM) are members of the PI3‐kinase like–kinase (PIKK) family of proteins. ATM is a well‐established tumour suppressor. Loss of one or both alleles of ATM results in an increased risk of cancer development, particularly haematopoietic cancer and breast cancer in both humans and mouse models. In mice, total loss of SMG1 is embryonic lethal and loss of a single allele results in an increased rate of cancer development, particularly haematopoietic cancers and lung cancer. In this study, we generated mice deficient in *Atm* and lacking one allele of *Smg1*, *Atm^−/−^Smg1^gt/+^* mice. These mice developed cancers more rapidly than either of the parental genotypes, and all cancers were haematopoietic in origin. The combined loss of *Smg1* and *Atm* resulted in a higher level of basal DNA damage and oxidative stress in tissues than loss of either gene alone. Furthermore, *Atm^−/−^Smg1^gt/+^* mice displayed increased cytokine levels in haematopoietic tissues compared with wild‐type animals indicating the development of low‐level inflammation and a pro‐tumour microenvironment. Overall, our data demonstrated that combined loss of *Atm* expression and decreased *Smg1* expression increases haematopoietic cancer development.

## 1 INTRODUCTION

SMG1 and ATM are members of the PI3‐kinase–like kinase (PIKK) family of proteins. Other members of this family include ATR, DNA‐PK_cs_ and mTOR. ATM is a well‐known tumour suppressor playing important roles in DNA damage repair and in responses to oxidative stress. In humans, loss of ATM causes the disease ataxia telangiectasia (A‐T) which is characterized by progressive neurodegeneration, immunodeficiency and cancer development; however, the progression of disease can be markedly different between individual cases.^1^ *Atm* knockout (−/−) mice are prone to the development of thymic lymphomas, are radiosensitive and show evidence of oxidative damage to tissues.^2^, ^3^, ^4^ Furthermore, loss of ATM and the resultant accumulation of unrepaired DNA damage can lead to activation of innate immune pathways and basal inflammation.^5^, ^6^ SMG1 has a well‐characterized role in nonsense‐mediated decay (NMD), the pathway used by cells to detect and degrade mRNA with premature termination codons which may code for truncated proteins.^7^ SMG1 has previously been implicated in regulation of DNA damage responses, telomere maintenance and oxidative and hypoxic stress responses and stress granule formation.^7^, ^8^, ^9^, ^10^, ^11^ Complete loss of SMG1 expression in mice is lethal early during embryogenesis but we have demonstrated that loss of a single SMG1 allele increased cancer development, particularly lung adenocarcinomas and lymphomas.^12^ SMG1 haploinsufficiency in these mice did not result in sufficient protein loss to affect its roles in nonsense‐mediated decay, DNA damage responses or apoptosis induction. However, SMG1‐deficient animals showed elevated levels of basal inflammation and oxidative damage to tissues prior to development of cancers indicating a potential role for these pathways in enhancing tumourigenesis in this model. SMG1 and ATM have previously been shown to co‐regulate DNA damage responses and p53 signalling.^13^, ^14^ Brumbaugh et al (2004) demonstrated that both enzymes contribute to the phosphorylation of Upf1 and p53 in response to ionizing radiation (IR). Further SMG1 and ATM are both required for maximal activation of the G1/S checkpoint following exposure to ionising radiation or during oxidative stress.^13^, ^14^ SMG1 can also regulate alternative splicing of p53 in response to DNA damage.^15^ To further examine the interplay between SMG1 and ATM in cancer development, we crossed mice heterozygous for both the *Smg1* genetrap allele (*Smg1^gt/+^*) and the *Atm* null allele to generate *Atm* knockout mice that were also heterozygous for *Smg1* (*Atm^−/−^Smg1^gt/+^*). These mice were viable and developed lymphomas at a more rapid rate than mice carrying only the *Atm^−/−^* or *Smg1^gt/+^* alleles.

## 2 MATERIALS AND METHODS

### 2.1 Generation of *Atm^−/−^Smg1^gt/+^* mice and animal husbandry

Parental strain *Atm^−/−^* and *Smg1^gt/+^* mice have been described previously.^12^, ^16^ Animals heterozygous for both alleles were bred to generate *Atm^−/−^Smg1^gt/+^* mice, and sex of parents carrying each genotype was alternated during breeding. All animal experiments were approved by the animal ethics committees of Western Sydney University, The University of Queensland or QIMR Berghofer Medical Research Institute and were conducted in accordance with the ‘Australian Code for the Care and Use of Animals for Scientific Purposes (2013)’. Mice were housed in either the QIMR Berghofer Medical Research Institute or Ingham Institute for Applied Medical Research animal facilities. Mice were kept on a 12:12 hour light: dark room cycle. Food and water were available ad libitum throughout the course of all experiments. Genomic DNA was extracted from ear clips for genotyping as described previously.^12^

### 2.2 Isolation and culture of mouse embryonic fibroblasts (MEFs)

Mating of mice was assumed at midnight and timed from 0.5. E14.5 embryos were isolated and brain and liver removed, and then trypsinized as described previously.^12^ MEFs were then plated on 0.1% gelatine‐coated T25 flasks and incubated at 37°C with 5% CO_2_ in DMEM (Thermo Fisher) supplemented with 12% heat‐inactivated foetal calf serum (Thermo Fisher), 1% penicillin/streptomycin (Thermo Fisher), 1% glutamax (Thermo Fisher), 1x non‐essential amino acid (Thermo Fisher) and beta‐mercaptoethanol (Sigma). Experiments on primary MEFs were performed at passages 4‐5.

### 2.3 Histology

Tissues were isolated and fixed in 10% formalin. Samples were processed by the QIMR Berghofer histology facility. Serial sections of 4μm thickness were used for haematoxylin and eosin staining or immunofluorescence for 8‐oxo‐dG or 4HNE as described previously.^17^ Images were scanned using Aperio Turbo or Aperio FL (Leica Biosystems) and analysed using ImageScope software (Leica Biosystems). Histology slides were examined by an experienced pathologist.

### 2.4 Irradiation and DNA damage analysis

Cells were irradiated at 6Gy with a GammaCell40Exactor (Best Theratronics Ltd.) with a Cobalt60 source of 960c Gγ/min. Cells were fixed with 4% paraformaldehyde in phosphate‐buffered saline (PBS) at indicated time‐points. γH2AX analysis by immunofluorescence was performed as described previously.^18^

### 2.5 Flow Cytometry—cell death assays, cytokine bead assays and cell surface markers

Flow cytometry was performed with a FACSCanto or Fortessa (BD Biosciences). Annexin V (BD Pharmingen) apoptotic analysis was performed according to manufacturer's instruction. Propidium iodide (Sigma) cell cycle analysis was performed as described previously.^19^ Measurement of serum cytokines levels was performed with cytokine bead array,^20^ and staining for cell surface markers was performed as described previously.^21^, ^22^

### 2.6 Quantitative PCR

Real‐time PCR reactions were performed as described previously 23 and analysed using an Applied Biosystems QuantStudio (Thermo Fisher). Primer sequences are shown in the table below.

**Table:** 

Target gene	Forward primer 5′‐3′	Reverse primer 5′‐3′	Ref
Interleukin‐1β	CAACCAACAAGTGATATTCTCCATG	GATCCACACTCTCCAGCTGCA	23
CSF‐1	CCACCATCCACTTGTATGTCAAAGAT	CTCAACCACTGTCACCTCCTGT	24
Rpl13a	GAGGTCGGGTGGAAGTACCA	TGCATCTTGGCCTTTTCCTT	25
Interleukin‐6	GATTGTATGAACAACGATGATGC	TGTTCTTCATGTACTCCAGGTAGC	12
IFNβ	CCACAGCCCTCTCCATCAAC	TGAAGTCCGCCCTGTAGGTG	23

### 2.7 Statistical analysis

Graphing and statistical evaluation was performed with GraphPad Prism 6 (GraphPad Software, USA). The Kaplan‐Meier function was used for survival curves and to estimate the median survival. Differences between survival curves were calculated using a log‐rank test. *t* Test with Welch's correction for unequal variance was used for all other comparisons. Differences were considered to be significant if *P* ≤ .05. Data are presented as mean ± standard error of the mean from at least three independent experiments unless otherwise indicated.

## 3 RESULTS

### 3.1 Generation of *Atm^−/−^Smg1^gt/+^* mice

The *Smg1* Genetrap line (*Smg1^gt/+^*) and *Atm* knockout mice (*Atm^−/−^*) have been described previously.^12^, ^16^ Animals heterozygous for both alleles were bred to generate *Atm^−/−^Smg1^gt/+^* mice. Animals were generated at approximately the expected frequency (1/7, when accounting for lethality of *Smg1* knockout animals).

### 3.2 **More rapid lymphoma development in** *Atm^−/−^* ***Smg1^gt/+^* mice**

**Figure 1:**
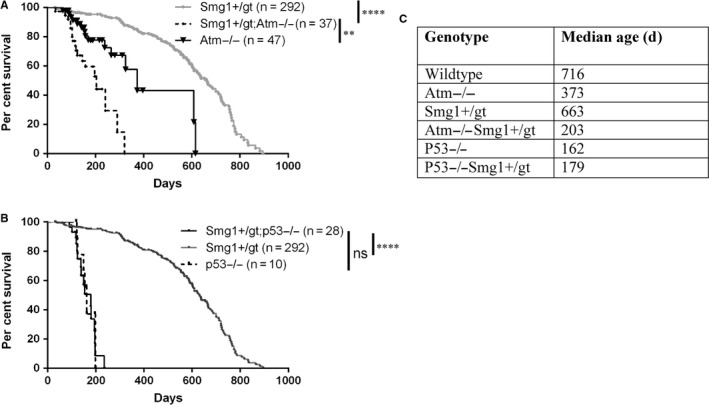
Combined *Atm* loss and *Smg1* heterozygosity decreases mouse lifespan. A, *Smg1^+/gt^* mice were bred with *Atm^+/−^* mice to generate *Smg1^+/gt^ Atm^−/−^* animals. Kaplan‐Meier survival curve shows that combined loss of *Atm* and *Smg1* significantly decreases lifespan. B, *Smg1^+/gt^* mice were also crossed to generate *Smg1^+/gt^p53^−/−^* animals. SMG1 heterozygosity had no additional effect on lifespan in these animals as demonstrated by Kaplan‐Meier survival curve. C, Median lifespan for each of the mouse lines examined as determined using Kaplan‐Meier analysis in GraphPad Prism. ***P < .001, ****P < .0001

*Atm^−/−^Smg1^gt/+^* mice were aged alongside littermate controls to examine spontaneous cancer development. *Atm^−/−^Smg1^gt/+^* mice had a significantly shorter lifespan than either *Atm^−/−^* or *Smg1^gt/+^* mice (Figure 1A&C). *Atm^−/−^Smg1^gt/+^* mice had a median lifespan of 203 days compared with 373 days for *Atm^−/−^* and 630 days for *Smg1^gt/+^* animals. At autopsy, *Atm^−/−^Smg1^gt/+^* animals were observed to have enlarged thymus and spleens. Tissue samples were taken and fixed in 10% formalin, then paraffin‐embedded, H&E‐stained and examined by a pathologist. All *Atm^−/−^Smg1^gt/+^* mice showed evidence of blood cancer development in either the spleen or thymus with small numbers of animals showing evidence of other pathologies such as steatosis, chronic inflammation or extramedullary haematopoiesis (Table 1). Further immunohistochemistry was performed on a subset of haematopoietic tumours to support pathology findings. Examples of results for IHC for B220 (B cell marker), MPO (myeloid marker), CD3 (T cell marker) and Bcl2 are in Figure S1. This cancer profile is more similar to the *Atm^−/−^* animals which also developed lymphomas in the majority of animals (80%, Table 1), in contrast *Smg1^gt/+^* mice who developed a combination of lymphomas and papillary lung adenocarcinomas as we have described previously (Table 1).^12^ We performed a similar experiment crossing *Smg1^gt/+^* mice to *p53^−/−^* mice. In contrast to the results with *Atm^−/−^* crosses, SMG1 haploinsufficiency had no effect on the rate of tumour development in *p53^−/−^* mice (Figure 1B‐C) nor on the types of tumours developed in *p53^−/−^Smg1^gt/+^* animals (Table 1). IHC was also performed with examples in Figure S2. These data indicate that decreased SMG1 expression exacerbates the pro‐oncogenic effect of ATM loss, potentially by increasing the dysregulation of a pathway in which both SMG1 and ATM provide regulatory feedback or by a combined effect on independently regulated pathways.

**Table 1:** Pathology results from tissues analysed from the different genotypes of mice

Tissue	Wild‐type (n = 10)	*Smg1^+/gt^* (n = 12)	*Atm^−/−^* (n = 8)	*Smg1^+/gt^ Atm^±^* (n = 8)	*Smg1^+/gt^ Atm^−/−^* (n = 16)	*Smg1^+/gt^ p53^−/−^* (n = 6)
Thymus		DLCL (4)	DLCL (2), lymphoblastic lymphoma (2)	FCL (1), lymphoma (1), large cell lymphoma (1)	Lymphoblastic lymphoma (2), DLCL (4), small cell lymphoma (1)	Lymphoma Burkitt‐like (4)
Liver	Large cell lymphoma (1), chronic inflammation (2)	Steatosis (3), DLCL (1), lymphoma (1), chronic inflammation (1)	Steatosis (3), lymphoblastic lymphoma (1), hepatitis (1)	Cavernous haemangioma (2), steatosis (2), spindle cell haemangioendothelioma (2), FCL (1), chronic inflammation (1)	Myeloid leukaemia (1), lymphoblastic lymphoma (1), DLCL (4), steatosis (1)	
Spleen	Large cell lymphoma (1), EMH (4), hyperplasia (1)	EMH (4), hyperplasia (1), DLCL (4), lymphoma (1)	DLCL (1), EMH (2), hyperplasia (1), lymphoblastic lymphoma (1),	FCL (1), DLCL (1), EMH (1), hyperplasia (1), hairy cell leukaemia (1)	Myeloid leukaemia (2), lymphoblastic lymphoma (2), DLCL(6), EMH (1), hyperplasia (1), marginal zone lymphoma (1)	EMH (1), FCL (1), hyperplasia (1)
Kidney	Chronic inflammation (1)	Chronic inflammation (3), DLCL(2), lymphoma (1)			Lymphoma (1), atypical lymphoid infiltrate(1), chronic inflammation (1)	
Lung	Chronic inflammation (1), lymphoid infiltrate (1)	Papillary adenocarcinoma (5), DLCL (1), chronic inflammation (2),	DLCL (2), papillary adenocarcinoma (1)	Chronic inflammation (1), papillary adenocarcinoma (5), FCL (1), DLCL (1)	Myeloid leukaemia (1), lymphoblastic lymphoma (1), DLCL(3), focal lymphoma (1)	Lymphoma Burkitt‐like (2)
Large Intestine					Myeloid leukaemia (1)	
Bone			Lymphoma (1)			
Heart		DLCL (1)			Lymphoma (1)	
Other	Large cell lymphoma (1)		Sarcoma (1)	Epidermal cyst (1)		High‐grade sarcoma (2)
Ovary				Serous cysts (1)		

Abbreviations: DLCL, Diffuse large cell lymphoma; EMH, extramedullary haematopoiesis; FCL, follicular cell lymphoma.

### 3.3 Effect of combined loss of SMG1 and ATM on haematopoietic cell composition

**Figure 2:**
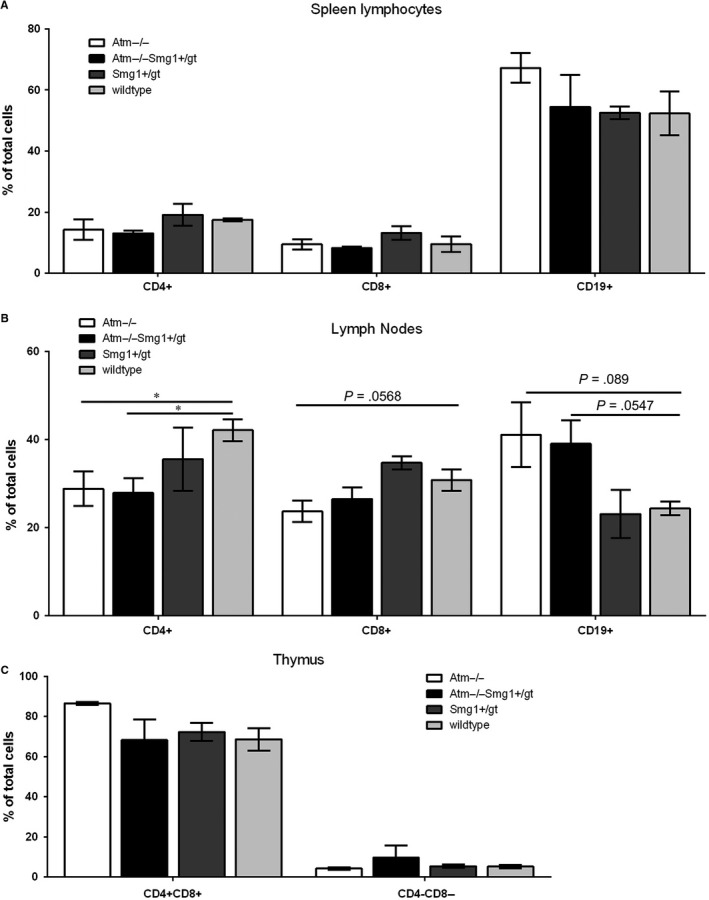
*Smg1* heterozygosity in addition to *Atm* loss does not alter lymphocyte profile compared with *Atm^−/−^* mice. Pre‐disease animals (3‐5 mo) were killed and spleen (A), lymph nodes (B) and thymus (C) harvested. Single‐cell suspensions were generated, cell surface marker staining was performed for major lymphocyte populations, and samples were analysed by flow cytometry. Bars show the mean and error bars the standard error of the mean. Statistical significance was assessed using a *t* test with Welch's correction for unequal variance. *P < .05

Given the predominance of lymphoma formation in *Atm^−/−^Smg1^gt/+^* mice, we examined whether the addition of *Smg1* heterozygosity resulted in increased defects in the immune system compared with the known decrease in T cells caused by loss of ATM expression.^2^, ^3^ We showed previously that SMG1 heterozygosity alone did not significantly alter the composition of immune system prior to disease onset.^12^ Here we analysed the composition of circulating blood cells and lymphocytes in the spleen, thymus and lymph nodes. There were no significant differences in the major circulating cell populations between any of the genotypes (Figure 2A). As expected *Atm^−/−^* animals showed a decreased number of both CD4+ and CD8+ T cells in spleen and lymph nodes (Figure 2B), this decrease was not affected by SMG1 heterozygosity. The percentage of CD19 positive B cells was increased in all animals lacking ATM expression in both the spleen and lymph node, and this is seen as the balance to the decreased percentage of T cells and is expected. In *Atm^−/−^* mice, there is an increased number of double‐positive T cells. ATM is important for repair of the DNA damage induced during VDJ recombination, and as such, there is a partial block in T cell differentiation in these animals. This increase was not observed in *Atm^−/−^Smg1^gt/+^* mice although there was an increase in double‐negative T cells which was not statistically significant (Figure 2C). As this difference does not translate to a difference in tissue T cell populations between *Atm^−/−^* and *Atm^−/−^Smg1^gt/+^* mice, it is unlikely to be contributing to tumour development differences between these animals.

### 3.4 Effect of *Smg1* haploinsufficiency on DNA damage responses and oxidative stress

**Figure 3:**
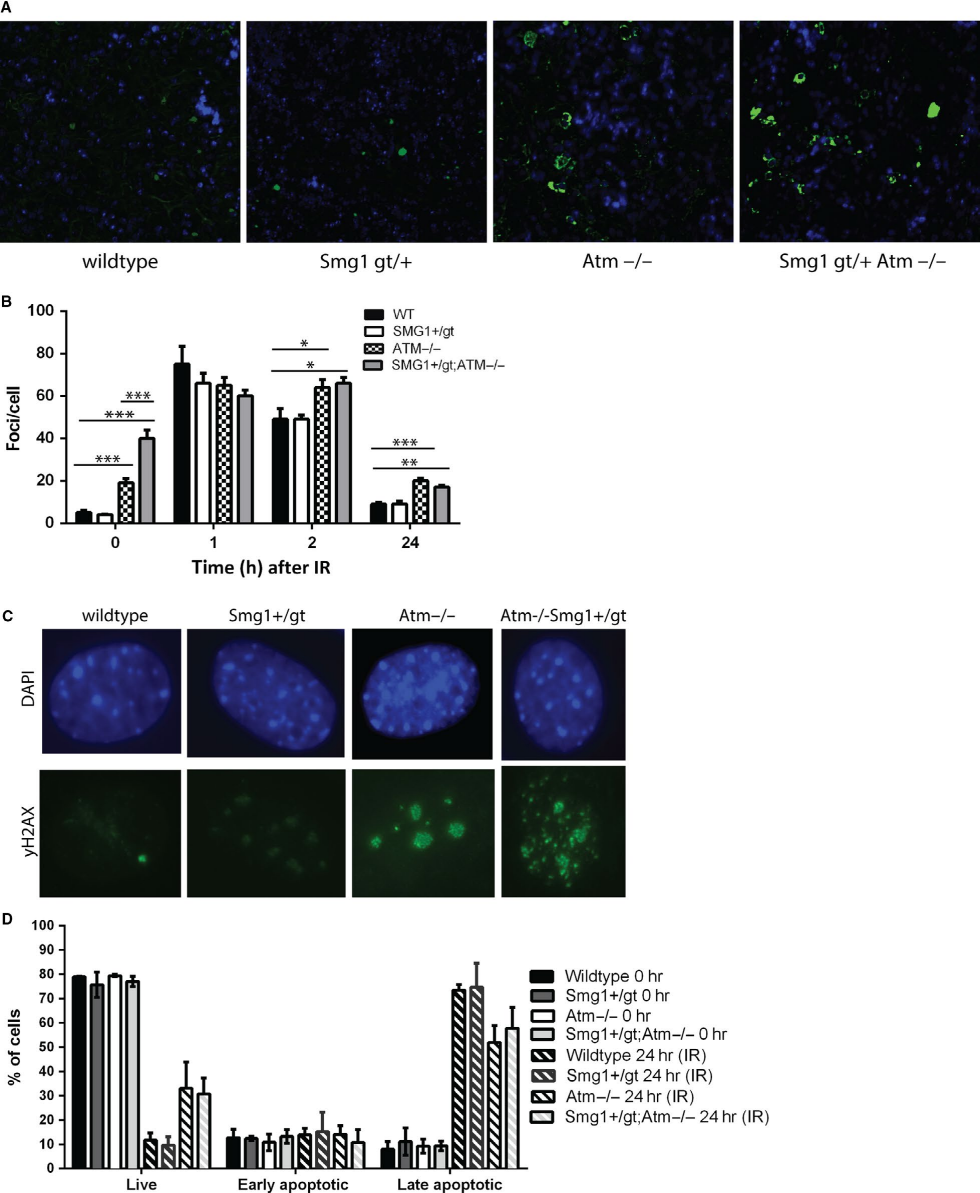
*Smg1* heterozygosity combined with *Atm* loss increases basal oxidative stress and DNA damage burden. A, *Smg1* heterozygosity combined with *Atm* loss increases oxidative damage to tissues. Spleens were harvested from pre‐disease animals, and immunofluorescence was performed for the marker of oxidative damage to DNA, 8‐oxo‐dG (green) and DAPI to highlight nuclei (blue). B, C, Murine embryonic fibroblasts were generated and exposed to 5 Gy irradiation (IR). Cells were fixed at the indicated time‐points post‐IR and stained for the presence of γH2AX as a marker of unrepaired DNA damage. The number of γH2AX foci in each cell nucleus was counted, and quantification is shown in panel A and example 0hr images in panel B γH2AX (green) and DAPI (blue). Statistical significance was determined using a *t* test with Welch's correction for unequal variance. Bars indicate the mean and error bars the standard error of the mean. *P < .05, **P < .01, ***P < .001. D, Thymocytes were isolated from mice and exposed to 5Gy irradiation (IR). At baseline and 24 h post‐IR, apoptosis was measured using AnnexinV/PI staining and flow cytometry. Bars indicate the mean and error bars the standard error of the mean

In both *Atm^−/−^* and *Smg1^gt/+^* mice, high levels of oxidative damage to tissues have been detected.^12^, ^16^ We examined tissues from *Atm^−/−^Smg1^gt/+^* mice and the parental genotypes to determine whether like DNA damage, oxidative damage was increased. Splenic tissue from pre‐disease mice was stained for the presence of 8‐oxo‐dG, a product of oxidative damage to DNA. Whereas there was limited detectable 8‐oxo‐dG signal in wild‐type animals, there was a low level of damage in *Smg1^gt/+^* mice as we have reported previously, this further increased in *Atm^−/−^* animals and again there was more damage detected in *Atm^−/−^Smg1^gt/+^* mice (Figure 3A). We further examined damage by staining splenic section for 4‐hydroxynonenal (4HNE) a product caused by lipid peroxidation in response to increased reactive oxygen or nitrogen species. 4HNE staining was also increased in *Atm^−/−^Smg1^gt/+^* mice to a greater degree than in either *Smg1^gt/+^* or *Atm^−/−^* animals (Figure S3). ATM has a well‐established role in the DNA damage response.^26^ We previously showed that the level of SMG1 protein expressed in *Smg1^gt/+^*mice was sufficient for its roles in DNA damage repair.^12^ However, others have shown that combined loss of ATM and SMG1 in cell lines leads to increased basal DNA damage and alterations to DNA repair pathways.^8^, ^14^ To determine whether the combined roles of SMG1 and ATM in DNA repair was recapitulated in vivo*,* we examined the formation and resolution of γH2AX foci in *Atm^−/−^* compared with *Atm^−/−^Smg1^gt/+^* murine embryonic fibroblasts (MEFs). γH2AX is recruited to sites of DNA double‐strand breaks and remains present at the break until it is repaired; thus, by measuring the changes in the number of foci we could determine whether the kinetics of DNA repair was altered by SMG1 haploinsufficiency on an *Atm^−/−^* background. MEFs were exposed to a moderate dose of 5Gy irradiation (IR), and the number of foci present in the nucleus of at least 50 cells was determined at baseline and 1, 2 and 24 hours post‐IR (Figure 3B). At baseline, it was clear that there was a larger number of γH2AX foci in cells from *Atm^−/−^* mice and that this was further increased in *Atm^−/−^Smg1^gt/+^* mice (Figure 3C example images). Following irradiation, a similar level of damage was observed in all genotypes but the rate of repair was slower in *Atm^−/−^* and *Atm^−/−^Smg1^gt/+^* compared with *Smg1^gt/+^* and wild‐type mice with a greater number of foci remaining at 2 and 24 hours post‐IR. Interestingly *Atm^−/−^Smg1^gt/+^* cells had a lower number of foci at 24 hours post‐IR than at baseline suggesting that IR may induce other DNA repair pathways to repair the damage that were not activated in the basal state in these cells. This was not the case for *Atm^−/−^* cells where numbers were similar to baseline. We also examined cell death in response to irradiation, thymocytes were isolated from mice and exposed to 5Gy irradiation, and apoptosis was determined by Annexin V/ PI staining and flow cytometry analysis. As expected from previous literature, *Atm^−/−^* thymocytes had a greater proportion of cells surviving at 24h (live) and a lower percentage of cells in late apoptosis than *Smg1^gt/+^* or wild‐type mice (Figure 3D). *Atm^−/−^Smg1^gt/+^* thymocytes showed no significant difference in cell death compared with *Atm^−/−^* cells. This makes sense given that once DNA repair pathways were induced the level of DNA damage retained in *Atm^−/−^Smg1^gt/+^* cells returned to the same level as *Atm^−/−^* cells (Figure 3B).

### 3.5 Effect of *Smg1* haploinsufficiency on systemic inflammation

**Figure 4:**
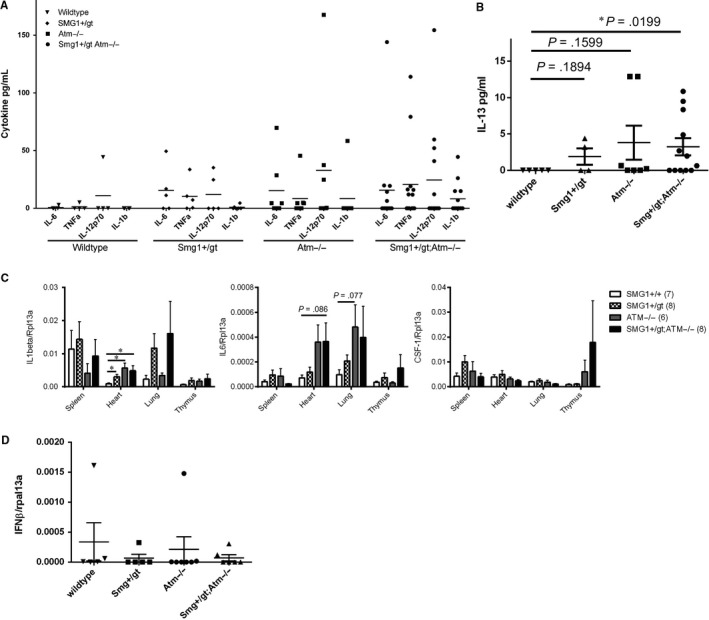
*Smg1* heterozygosity combined with *Atm* loss may increase basal inflammation. A, B, Serum was isolated from mice and cytokine levels analysed by cytokine bead assay. Symbols show the value for each individual animal, and bars show the average. In panel B, error bars show the standard error of the mean. C, Spleen, heart, lung and thymus were harvested from pre‐disease animals, RNA was isolated, and cytokine mRNA levels were measured by quantitative PCR. Bars show the mean and error bars the standard error of the mean. D, IFNβ mRNA levels were measured in spleen samples from pre‐disease animals by quantitative PCR. Symbols show the results for individual animals, horizontal lines the average and error bars the standard error if the mean. Statistical significance was determined using a *t* test with Welch's correction for unequal variance. *P < .05

Our previous investigations showed that *Smg1^gt/+^* mice had elevated levels of tissue and serum cytokines prior to the onset of disease.^12^ Furthermore, loss of ATM can result in increased inflammation due to unrepaired DNA damage resulting in an accumulation of cytosolic DNA and subsequent pro‐inflammatory cytokine production.^5^, ^17^ As an inflammatory microenvironment may also contribute to the development of haematopoietic tumours, we determined cytokine levels in *Atm^−/−^Smg1^gt/+^* mice. Animals were killed between 3 and 5 months of age, and tissues and serum were collected. Cytokine levels were measured by real‐time PCR and CBA assay. In serum samples, cytokine levels were highly variable between individuals. For a subset of NF‐κB–dependent cytokines (interleukin‐6 [IL‐6], tumour necrosis factor α [TNFα], IL‐1β and IL‐12p70), levels were generally undetectable in wild‐type mice and we could observe a small difference in cytokine levels between wild‐type and *Smg1^gt/+^* mice, though a smaller magnitude difference than we had observed previously (Figure 4A). This is likely due to the earlier sacrifice time of the animals in this study compared with 6‐9 months in our original study.^12^ The number of animals with higher levels of cytokines increased in *Atm^−/−^* mice and *Atm^−/−^Smg1^gt/+^* mice although the difference in level was not significantly different due to the variability between individuals (Figure 4A). However, it appears that mice lacking *Atm* and especially when combined with *Smg1* heterozygosity are more likely to express higher levels of these cytokines. Also of interest was the pattern of expression of IL‐13. IL‐13 was undetectable in wild‐type and expressed at low levels in *Smg1^gt/+^* mice, two *Atm^−/−^* mice expressed high levels of IL‐13, and half of the *Atm^−/−^Smg1^gt/+^* mice expressed detectable IL‐13 levels (Figure 4B). As serum levels provide an overview of systemic inflammation, we also measured IL‐1β, IL‐6 and colony‐stimulating factor 1 (CSF‐1) levels by quantitative PCR (Figure 4C). The only significant differences in cytokine expression were IL‐1β expression in the heart with *Smg1^gt/+^*, *Atm^−/−^* and *Atm^−/−^Smg1^gt/+^* mice all having significantly elevated levels compared with wild‐type. IL‐6 in the heart and lung appeared to be increased in *Atm^−/−^* and *Atm^−/−^Smg1^gt/+^* mice but this did not reach statistical significance. We also measured the levels of IFNβ in splenic tissues but this was undetectable in nearly all samples (Figure 4D).

## 4 DISCUSSION

Both SMG1 and ATM have been shown previously to act as tumour suppressors.^12^, ^27^, ^28^, ^29^ Here we demonstrated that loss of one of the alleles of *Smg1* in addition to *Atm* loss resulted in more rapid cancer development. Also all cancers arose from the haematopoietic system in contrast to either *Smg1^+/gt^* or *Atm^−/−^* mice where there were a large proportion of haematopoietic cancers but solid cancers, particularly lung adenocarcinomas, were also prevalent 12, 16 (Table 1). Although the combined loss of *Smg1* and *Atm* did not alter the composition of the immune system compared with loss of *Atm* alone (Figure 2), it did result in a significant increase in basal DNA damage load and oxidative stress (Figure 3). In response to induced DNA damage, SMG1 and ATM can act in concert to regulate p53 phosphorylation and G1/S checkpoint activation in response to DNA damage.^13^, ^14^ When *Atm* or *Smg1* are knocked out, it results in cells continuing to proliferate even in the presence of unrepaired DNA damage, failing to be blocked in their passage through the cell cycle at the G1/S phase transition, often referred to as radioresistant DNA synthesis.^13^, ^30^ This is due to defective p53 phosphorylation, in the absence of ATM at the G1/S checkpoint. SMG1 can also control p53 activation by regulating the expression of alternatively spliced p53 isoforms.^15^ Further loss of either *Smg1* or *Atm* alone results in increased oxidative stress and the resultant damage to DNA.^2^, ^12^ As such when we combined loss of *Atm* and *Smg1* in *Atm^−/−^Smg1^gt/+^* mice we saw exacerbated oxidative damage in tissues and increased basal DNA damage load. Given that increased damage burden did not result in greater cell death in *Atm^−/−^Smg1^gt/+^* cells (Figure 3C), the combined data suggest that cells are surviving but are more likely to develop an oncogenic mutation, due to low level DNA damage, which could contribute to the development of cancers in these animals.

ATM and SMG1 have also both been implicated in the regulation of inflammation with loss increasing basal cytokine levels (reviewed in 31). A low‐level, continuous inflammatory response or ‘smouldering’ inflammation is a pro‐tumourigenic microenvironment.^32^ This is particularly true for haematopoietic cancers where the cytokines can also act directly as growth factors; for example, increased IL‐6 drives B cell growth and proliferation via activation of STAT3.^33^ Along with STAT3, NF‐κB is a key transcription factor which can drive tumourigenesis and support cancer cell survival.^34^ In *Atm^−/−^Smg1^gt/+^* mice, we saw a trend of increasing levels of NF‐κB–dependent cytokine expression indicating that there was ongoing NF‐κB activation in these animals (Figure 4A and 4). This was not consistent across all animals at a given time‐point but this may reflect the range of times at which *Atm^−/−^Smg1^gt/+^* mice develop cancer. All our cytokine analysis was performed in pre‐disease animals (3‐5 months of age showing no signs of disease or enlargement of haematopoietic organs upon sacrifice) based on the survival curve (Figure 1A). Some of these animals would have been much closer in time to developing cancers than others, and as such, only a proportion of mice had detectable tumour promoting cytokines at this time‐point. Interestingly, we also saw a significant increase in IL‐13 levels in the serum in *Atm^−/−^Smg1^gt/+^* mice compared with wild‐type littermates (Figure 4B). IL‐13 also has a role in creating a pro‐tumour environment via the activation of tumour‐associated macrophages and myeloid‐derived suppressor cells.^35^ Together these data suggest that as they age *Atm^−/−^Smg1^gt/+^* mice may more quickly develop a pro‐tumour microenvironment compared with control animals.

What was not increased in *Atm^−/−^Smg1^gt/+^* mice was type I interferon production. Long‐term unrepaired DNA damage results in accumulation of DNA in the cytoplasm of cells which activates the cGAS/STING pathway to induce interferon (IFN) production.^36^ This has previously been demonstrated in bone marrow‐derived cells from *Atm^−/−^* mice and in isolated cells and tissues from rats lacking ATM expression.^5^, ^6^, ^17^ However, in tissues analysed here IFNβ was barely detected in any *Atm^−/−^Smg1^gt/+^* mice (Figure 4D). At this same time–point, increased oxidative DNA damage was evident in spleen (Figure 3A) but it is possible that this had not persisted for long enough to lead to IFN induction. Alternatively, loss of SMG1 in addition to ATM loss may limit IFNβ production by an unknown mechanism.

Overall, our data demonstrate that combined loss of expression of ATM and heterozygosity of SMG1 results in more rapid cancer development particularly haematopoietic cancers. Pre‐disease animals showed increased unrepaired DNA damage and oxidative stress and propensity to develop “smouldering” inflammation most clearly in haematopoietic tissues. The combination of persistent DNA damage and a permissive tumour microenvironment represents a likely mechanism resulting in increased and more rapid development of blood cancers. In total, this work further highlights the extensive crosstalk and dual regulation of pathways by members of the PIKK family.

## CONFLICT OF INTEREST

The authors declare no conflicts of interest.

## AUTHOR CONTRIBUTION

UH, JL, AJ, CSL, HQ, HCL, SA, YCL and TLR performed experimentation and analysed data; MFL, UH and TLR designed experiments; and UH and TLR wrote the manuscript with feedback from all authors.
