# Supplementary figures and images for: SMG1 heterozygosity exacerbates haematopoietic cancer development in Atm null mice by increasing persistent DNA damage and oxidative stress
Source: J Cell Mol Med. 2019 Sep 29;23(12):8151–60. doi: 10.1111/jcmm.14685 (PMC6850945; doi:10.1111/jcmm.14685)

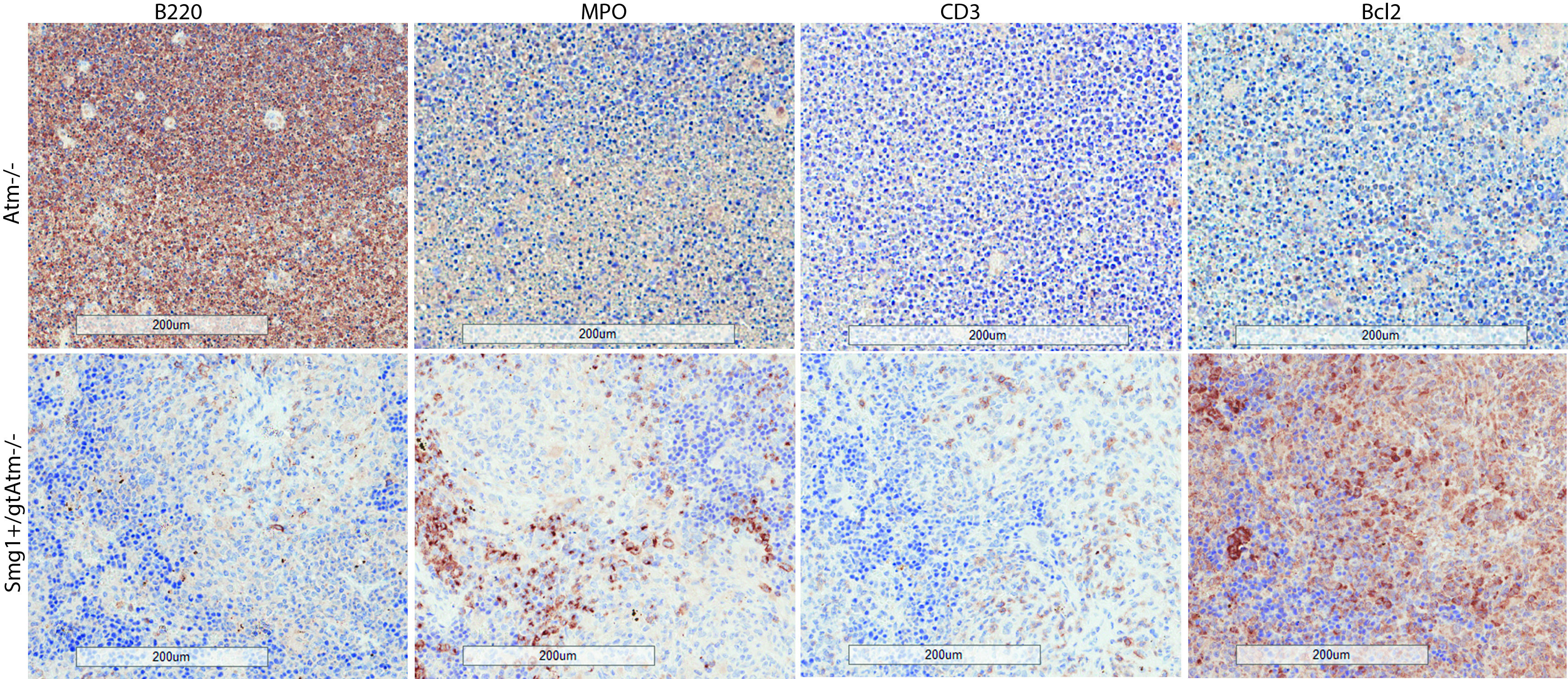

Supplement: Supplementary file 1 [file JCMM-23-8151-s001.jpg]

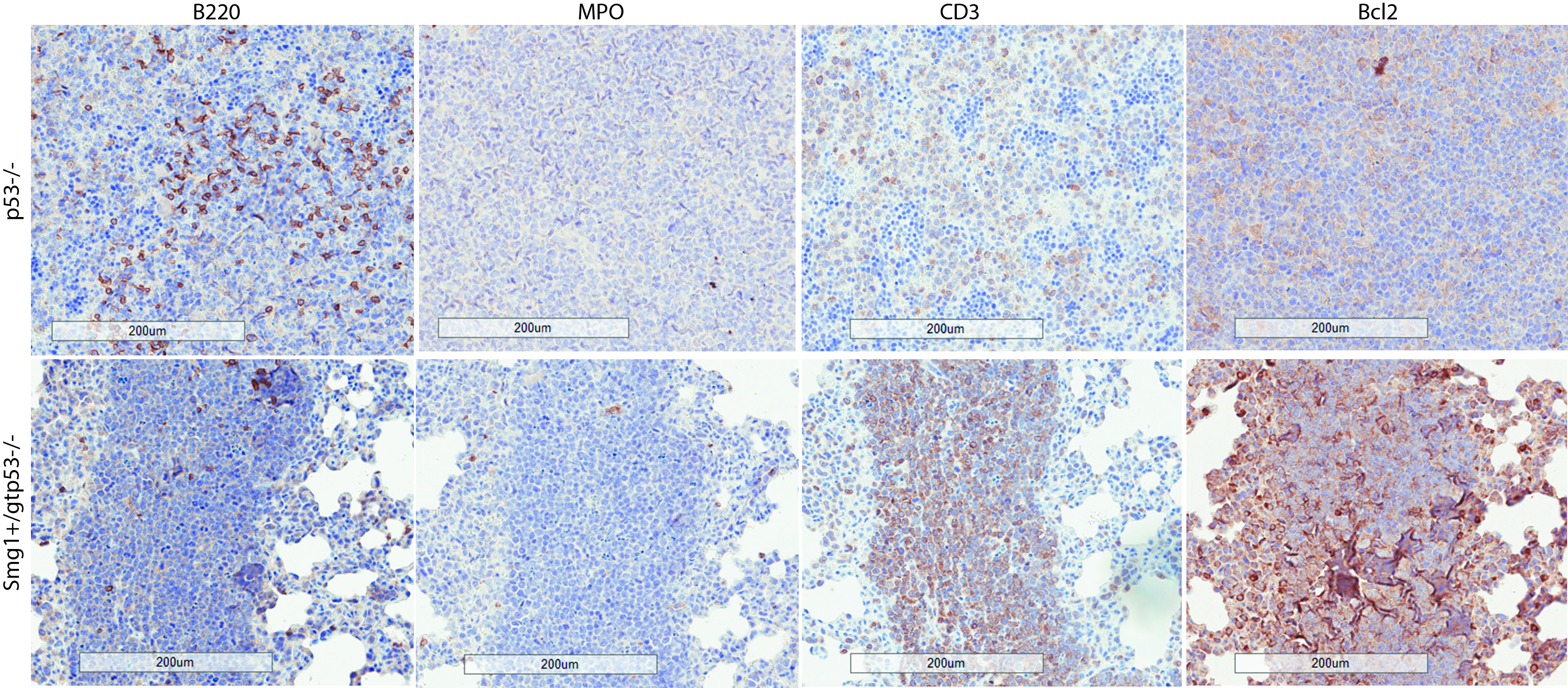

Supplement: Supplementary file 2 [file JCMM-23-8151-s002.jpg]

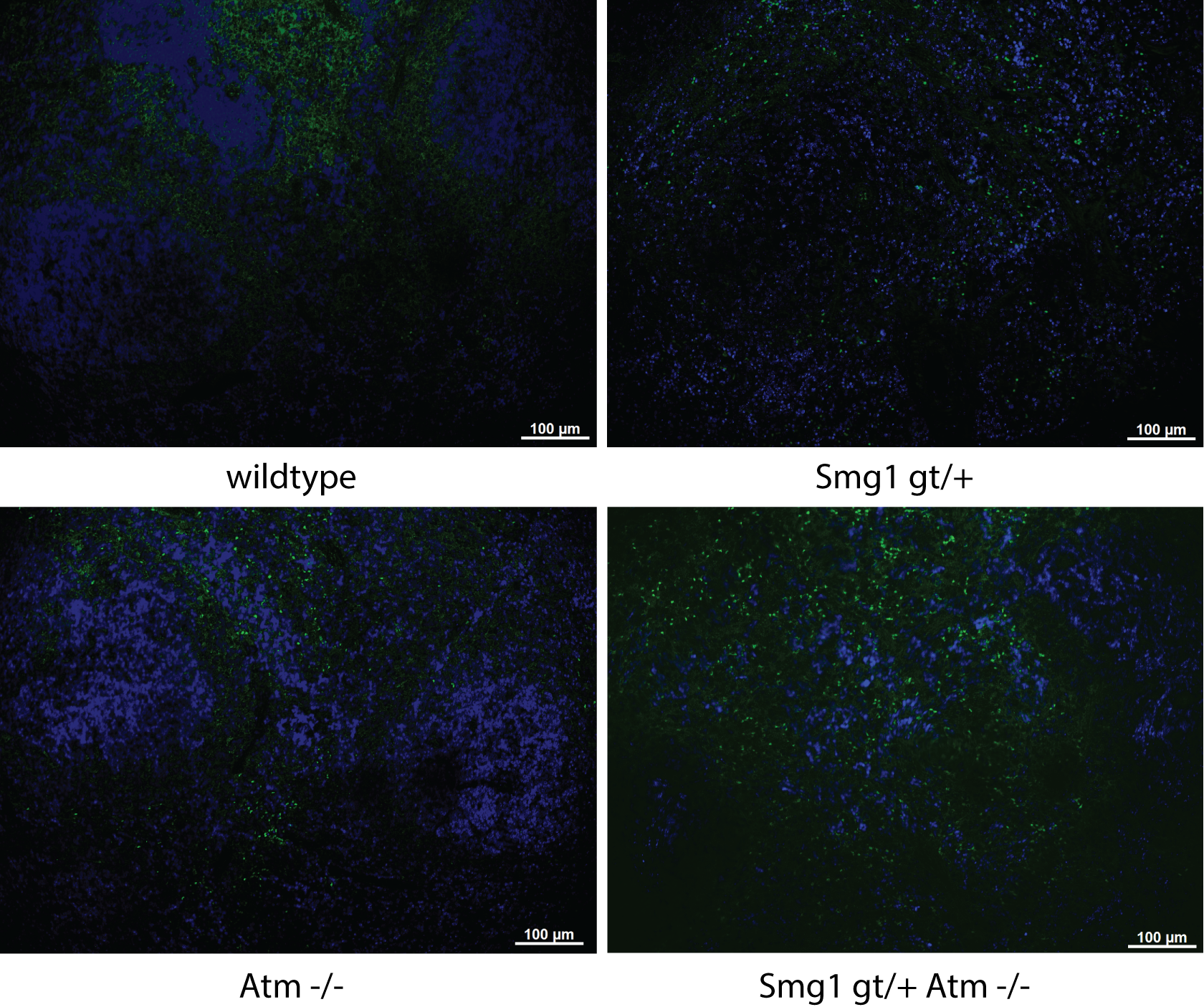

Supplement: Supplementary file 3 [file JCMM-23-8151-s003.tif]
